# A Novel Zebrafish Model to Provide Mechanistic Insights into the Inflammatory Events in Carrageenan-Induced Abdominal Edema

**DOI:** 10.1371/journal.pone.0104414

**Published:** 2014-08-20

**Authors:** Shi-Ying Huang, Chien-Wei Feng, Han-Chun Hung, Chiranjib Chakraborty, Chun-Hong Chen, Wu-Fu Chen, Yen-Hsuan Jean, Hui-Min David Wang, Chun-Sung Sung, Yu-Min Sun, Chang-Yi Wu, Wangta Liu, Chung-Der Hsiao, Zhi-Hong Wen

**Affiliations:** 1 Department of Marine Biotechnology and Resources, National Sun Yat-sen University, Kaohsiung, Taiwan; 2 Center for Neuroscience, National Sun Yat-sen University, Kaohsiung, Taiwan; 3 Department and Graduate Institute of Aquaculture, National Kaohsiung Marine University, Kaohsiung, Taiwan; 4 Doctoral Degree Program in Marine Biotechnology, National Sun Yat-sen University and Academia Sinica, Kaohsiung, Taiwan; 5 Department of Neurosurgery, Kaohsiung Chang Gung Memorial Hospital and Chang Gung University College of Medicine, Kaohsiung, Taiwan; 6 Department of Orthopaedic Surgery, Ping-Tung Christian Hospital, Ping-Tung, Taiwan; 7 Department of Fragrance and Cosmetic Science, Kaohsiung Medical University, Kaohsiung, Taiwan; 8 Graduate Institute of Natural Products, Kaohsiung Medical University, Kaohsiung, Taiwan; 9 Department of Anesthesiology, Taipei Veterans General Hospital, Taipei, Taiwan; 10 School of Medicine, National Yang-Ming University, Taipei, Taiwan; 11 Department of Biological Sciences, National Sun Yat-sen University, Kaohsiung, Taiwan; 12 Department of Biotechnology, and Center for Infectious Disease and Cancer Research, Kaohsiung Medical University, Kaohsiung, Taiwan; 13 Department of Bioscience Technology, Chung Yuan Christian University, Chung-Li, Taiwan; 14 Center of Nanotechnology, Chung Yuan Christian University, Chung-Li, Taiwan; National University of Singapore, Singapore

## Abstract

A suitable small animal model may help in the screening and evaluation of new drugs, especially those from natural products, which can be administered at lower dosages, fulfilling an urgent worldwide need. In this study, we explore whether zebrafish could be a model organism for carrageenan-induced abdominal edema. The research results showed that intraperitoneal (i.p.) administration of 1.5% λ-carrageenan in a volume of 20 µL significantly increased abdominal edema in adult zebrafish. Levels of the proinflammatory proteins tumor necrosis factor-α (TNF-α) and inducible nitric oxide synthase (iNOS) were increased in carrageenan-injected adult zebrafish during the development of abdominal edema. An associated enhancement was also observed in the leukocyte marker, myeloperoxidase (MPO). To support these results, we further observed that i.p. methylprednisolone (MP; 1 µg), a positive control, significantly inhibited carrageenan-induced inflammation 24 h after carrageenan administration. Furthermore, i.p. pretreatment with either an anti-TNF-α antibody (1∶5 dilution in a volume of 20 µL) or the iNOS-selective inhibitor aminoguanidine (AG; 1 µg) inhibited carrageenan-induced abdominal edema in adult zebrafish. This new animal model is uncomplicated, easy to develop, and involves a straightforward inducement of inflammatory edema for the evaluation of small volumes of drugs or test compounds.

## Introduction

Inflammation, a complex reaction of the immune system, is caused by multiple biological responses in tissues and can occur in all types of tissue during injury. This process is part of the non-specific immune response. Certain physiological symptoms, such as increased blood flow, elevated cellular metabolism, vasodilatation, the release of soluble mediators, the accumulation of fluid, and cellular influx are the hallmarks of an inflammatory response. However, in some disorders, the normal inflammatory process is prolonged and contributes to the development of chronic inflammatory diseases [1]. Inflammatory cells are recruited to the site of inflammation by proinflammatory mediators such as cytokines, chemokines, etc. [2]. Edema, an abnormal accumulation of fluid in the tissue, may develop due to inflammation. It may occur in specific organs or in any part of the body. Abdominal edema is a common form of inflammation that occurs in the abdomen and causes inflammatory swelling. Without proper treatment, the situation can become severe and may even cause death [3]. The cellular basis of abdominal edema and its molecular mechanisms are not fully understood. Therefore, it is necessary to study the molecular and cellular causes of abdominal edema and examine the underlying inflammatory mechanisms.

Starting in 1962, a rat-based inflammatory model, in which carrageenan was injected into a rat's paw, was developed to study inflammatory mechanisms. This classical inflammatory animal model has helped to investigate both inflammatory mechanisms and the anti-inflammatory activities of drugs [4]–[7]. Subsequently, a carrageenan-injected mouse model was developed to study the inflammatory process of paw edema [5], [8], followed by a guinea pig model [9]–[12]. However, it can be difficult to investigate anti-inflammatory drugs using these animal models because milligram amounts are needed to test the anti-inflammatory activity *in vivo* via systemic injection, especially in rodents [6], [7], and often there is not enough of the compounds left after screening with *in vitro* systems to examine bioactivity *in vivo*. It can be even more difficult to obtain sufficient quantities of the compounds for *in vivo* tests if the compounds have been derived from natural products. However, this problem can be solved using a zebrafish (*Danio rerio*) animal model. The use of zebrafish as an animal model has fewer limitations than the use of rodents, in terms of breeding, economy, efficiency, and low dosage [13], [14]. The size of the adult zebrafish is very small (approximately 4 cm in length); therefore, it also has a very low body weight. As a result, the amount of test compound required (which is calculated per gram body weight) is considerably lower than that required for other animal models, which is one of the most significant advantages of using zebrafish to screen new anti-inflammatory drugs.

Of late, the zebrafish is increasingly used in research, and has become a valuable animal model for drug discovery and development. Zebrafish have been used to screen bioactive compounds [15] and also in a wide variety of experimental studies to understand the pathogenic process underlying several immunological diseases [16], including the molecular mechanisms [13], [14], [17]. There have also been some studies in which the development of a zebrafish model of inflammation was attempted [14], [18]–[25]. However, the existing zebrafish inflammation model is still inadequate for the evaluation of bioactive compounds for 2 main reasons: First, no previous studies have assessed the typical external symptoms of inflammation, such as swelling, in zebrafish. Second, only a few previous studies have examined the modulatory effect of clinical agents with anti-inflammatory properties, such as steroids, on the inflammatory mediators in zebrafish during inflammation [14], [22], [25]–[27].

Tumor necrosis factor-α (TNF-α) and inducible nitric oxide synthase (iNOS) have been reported to play key roles in the edematous process and the inflammatory response. However, very few studies have demonstrated elevated protein expression of TNF-α and iNOS in carrageenan-induced inflamed paws in rodents [7], [28]–[30]. Using mouse or rat models, the roles of antibody-mediated TNF-α blockade and iNOS inhibition (using N-iminoethyl-L-lysine and aminoguanidine (AG)) in the inflammatory process have been understood [28]–[31], and thus far, no study has used a zebrafish model to evaluate carrageenan-induced inflammatory process.

In this study, we use a zebrafish model of carrageenan-induced inflammation to understand the inflammatory responses culminating in abdominal edema and the possible underlying mechanisms, and also examine the proinflammatory proteins released during the inflammation. We evaluated abdominal edema and proinflammatory proteins (TNF-α and iNOS) after intraperitoneal (i.p.) administration of carrageenan in adult zebrafish. We also measured myeloperoxidase (MPO), a leukocyte marker [32]–[34], to assess the level of tissue inflammation. In order to test the feasibility of using carrageenan-injected adult zebrafish as an inflammation model, we investigated the effects of the systemic administration of methylprednisolone (MP) (the positive control), an anti-TNF-α antibody, and AG on carrageenan-induced inflammatory responses.

## Materials and Methods

### 1. Chemicals

We obtained MS-222 (Tricaine), λ-carrageenan, MP, and AG from Sigma Co., Ltd. (St Louis, MO, USA).

### 2. Fish and Ethical Statement

Male AB strain adult zebrafish were obtained from Taiwan (Taikong Co., Taiwan), and the fish were housed within a temperature-controlled (28±1°C) laboratory aquarium system (Taikong Co., Taiwan) on a 14 h/10 h light/dark schedule as described by Westerfield [35]. All the experimental protocols were reviewed and approved by National Sun Yat-sen University Animal Care and Use Committee. We followed the Guiding Principles in the Care and Use of Animals of the American Physiology Society during zebrafish maintenance and experimentation. We also followed international laws and regulations for experiments in which live animals were used. All measurements were performed under anesthesia. The experimental design and execution was optimized to minimize the number of adult zebrafish used and their suffering.

### 3. Carrageenan injection in adult zebrafish and edema analysis

To induce inflammation in adult zebrafish, carrageenan was i.p. injected in a volume of 20 µL in phosphate-buffered saline (PBS) under MS-222 (168 ppm) anesthesia.

#### (1) Rat paw volume meter method

**Figure 1:**
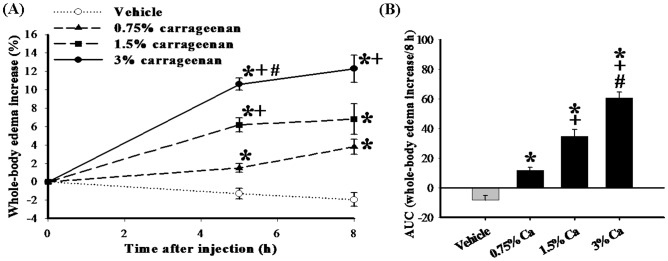
The dose responses for the whole-body edematous effects of carrageenan in adult zebrafish. Time course and the percentage change of whole-body volume induced by i.p. injection of vehicle (PBS) or carrageenan in adult zebrafish (A), using a rat paw volume meter. The basal whole-body volume of each adult zebrafish before i.p. injection of vehicle or carrageenan injection was considered as 100%, and the increase in whole-body volume is presented as a percentage change from the basal values by subtracting the basal whole-body volume from the whole-body volume measured at each time point. The absolute basal whole-body volume of adult zebrafish before the injection of vehicle or carrageenan is 593.9±49.3 µL (*n* = 36). The area under the abdominal-edematous-effect time curve (B), which was calculated from Figure A, for i.p. vehicle or carrageenan (0.75%, 1.5%, and 3%) injection extended from 0 to 8 h after i.p. carrageenan injection and showed that i.p. 0.75–3% carrageenan induced a significant increase in the whole-body volume in adult zebrafish. Each point or bar in all figures represents the mean ± SEM of 9 adult zebrafish per group. AUC: area under the curve; 0.75% Ca: 0.75% carrageenan; 1.5% Ca: 1.5% carrageenan; 3% Ca: 3% carrageenan; * *P*<0.05 compared with the same time points after i.p. injection of the vehicle (PBS) group; +*P*<0.05 compared with the same time points after i.p. injection of the carrageenan (0.75%) group; #*P*<0.05 compared with the same time points after i.p. injection of the carrageenan (1.5%) group.

We used the modified method described in our previous studies [6], [7]. After anesthesia, water was quickly removed from the body surface with a cotton swab. We measured zebrafish whole-body volume using a rat paw volume meter (plethysmometer; Singa Technology Corporation, Taiwan) and calculated changes in whole-body volume after carrageenan injection by subtracting the basal whole-body volume (before i.p. carrageenan injection) from the whole-body volume measured at each time point after injection. The graphical representation of abdominal edema versus time (Fig. 1B) was computed by the trapezoidal method [36] from 0 to 8 h after i.p. injection of vehicle (PBS) or carrageenan.

#### (2) Photographic image analysis method

**Figure 2:**
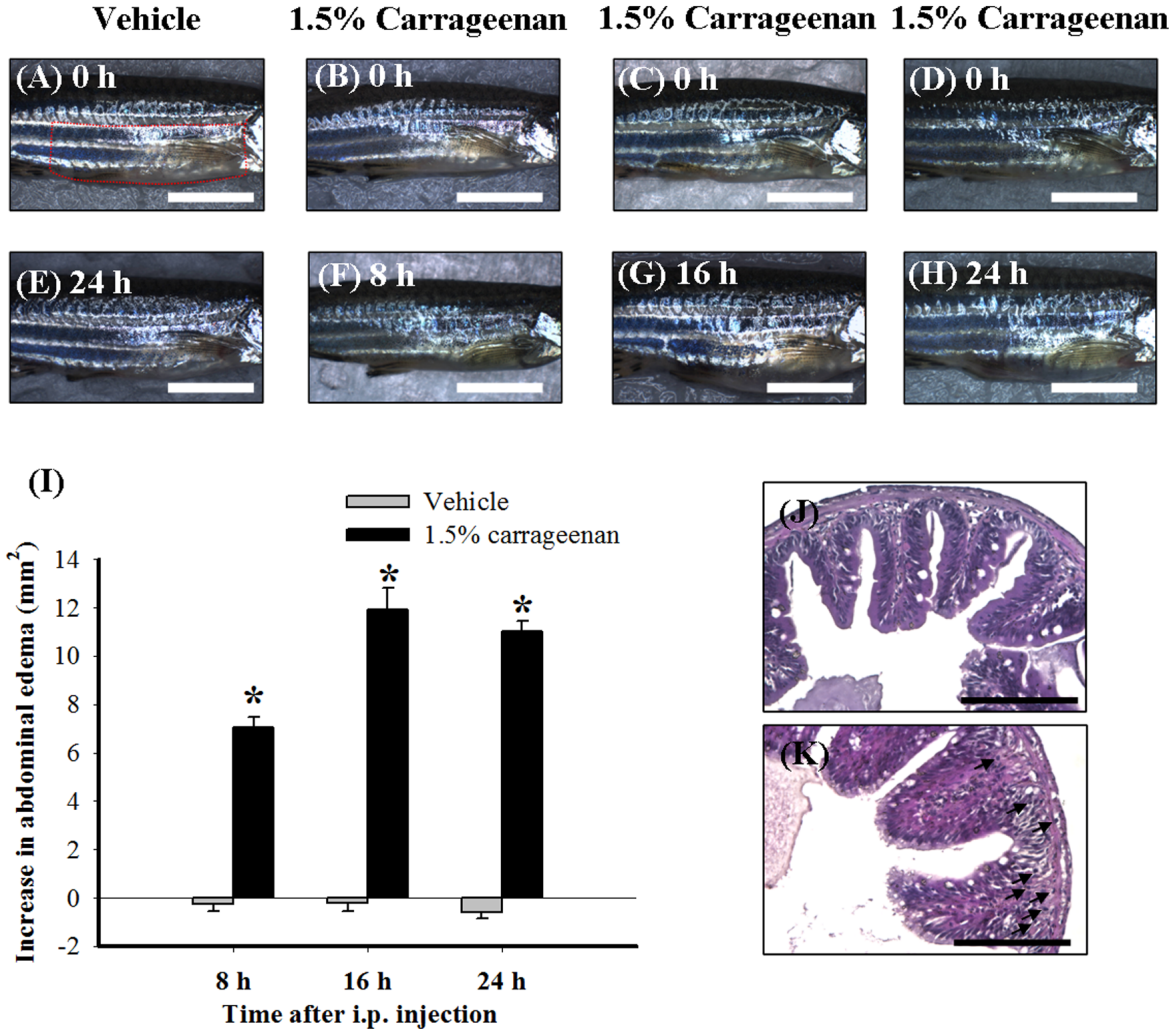
The time course for the abdominal-edematous effects of i.p. carrageenan (1.5%) in adult zebrafish. The photographic images show the gross pathology of the abdomen in the lateral view following i.p. injection of vehicle (PBS) plus i.p. injection vehicle (PBS) (A and E) and i.p. injection vehicle plus i.p. injection of 1.5% carrageenan at each time point (B–D and F–H). Images A–D were taken at 0 h after i.p. injection of vehicle or 1.5% carrageenan; images E, F, G, and H were taken at 24 h after i.p. injection of vehicle and 8, 16, and 24 h after 1.5% carrageenan injection. Scale bars: 5 mm for images A–H. The increase of the lateral area of the abdomen was given as a difference change from the basal values by subtracting the basal lateral area from the lateral area measured at each time point. Time course of change of the lateral area of abdomen induced by i.p. injection of vehicle (PBS) or carrageenan in adult zebrafish using a photographic image analysis system (I). Compared with the i.p. vehicle groups, the lateral area of abdomen increased progressively in the i.p. 1.5% carrageenan groups until 24 h. Sections (2 µm) of zebrafish abdominal tissues at 24 h after an i.p. injection of vehicle (J) or carrageenan (K). I.p. carrageenan dramatically induced leukocyte infiltration (arrows) in the intestine. Scale bars: 100 µm for images (J) and (K). Each bar in Figure (I) represents the mean ± SEM of 9 adult zebrafish per group. *P<0.05 compared with the same time points in the i.p. vehicle (PBS) group.

After anesthesia, to examine the gross pathology, abdominal imaging of adult zebrafish was conducted using a Leica Z16 APO macroscope (Leica Instruments Inc., Wetzlar, Germany) combined with a SPOT digital camera system (Idea 5 MP CMOS; SPOT Imaging Solutions, a division of Diagnostic Instruments, Inc., Sterling Heights, MI, USA). The lateral area (mm^2^) of the abdominal region was measured using SPOT Imaging software (SPOT Imaging Solutions, a division of Diagnostic Instruments, Inc., Sterling Heights, MI, USA) and defined by the following criterion: a straight line was drawn from the starting point at the base of the anal fin toward the lateral line, then along the lateral line until the operculum, then toward the abdomen, and then along the abdominal edge until the starting point (example shown in Fig. 2A). Changes in the lateral area of the abdominal region were calculated by subtracting the basal lateral area of the abdomen (before i.p. carrageenan injection) from the lateral area of abdomen measured at each time point after i.p. carrageenan injection.

### 4. Histopathology

For histopathological studies, adult zebrafish were first fixed in 10% neutral buffered formalin, soaked in decalcifying solution for 48 h, and then stocked in 10% formalin. For dehydration, clearing, and infiltration, abdominal tissues were placed in embedding cassettes using an automatic tissue processor (Tissue-Tek, Sakura Finetek Japan Co., Ltd, Japan), then embedded into paraffin blocks with a tissue embedding center (EC780-1; EC780-2, CSA). Finally, the tissue was cut into 2 µm sections using a rotary microtome (HM340E, Microm). For histopathological examination, abdominal sections were stained using hematoxylin and eosin (H&E) and then analyzed using a Leica DM-6000 CS microscope (Leica Instruments Inc., Wetzlar, Germany) and a microscope digital camera system (SPOT Idea 5 MP CMOS scientific color digital camera system, Diagnostic Instruments, Inc., Sterling Heights, MI, USA).

### 5. Western blot analysis for MPO, TNF-α, and iNOS

For western blot analysis, abdominal samples (all abdominal organs) from adult zebrafish were collected at specific time points, washed with ice-cold PBS, and homogenized in ice-cold lysis buffer (50 mM Tris, pH 7.5, 150 mM NaCl, 1 µg/mL aprotinin, 1% Triton X-100, 100 µg/mL phenylmethylsulfonyl fluoride) with a Bertin Precellys 24 homogenizer (Bertin Technologies, Aix-en-Provence, France) at 5500 rpm for 4 times for 20 s at 4°C. Then, samples were centrifuged at 20,000× g at 4°C for 60 min. We collected the supernatants for western blot analysis of MPO, TNF-α, and iNOS. For protein measurement, we used a modification of the method described by Lowry et al. [37], and protein concentrations in the supernatants were determined using the DC protein assay kit (Bio-Rad, Hercules, CA, USA). An equal volume of sample buffer (2% sodium dodecyl sulfate (SDS), 10% glycerol, 0.1% bromophenol blue, 50 mM Tris–HCl, pH 7.2, and 2% 2-mercaptoethanol) was added to each supernatant, which was then electrophoresed through a tricine SDS-polyacrylamide gel at 150 V for 90 min. We transferred the proteins in the gel (125 mA overnight at 4°C) to a polyvinylidene difluoride membrane (PVDF membrane; Immobilon-P, Millipore, 0.45-µM pore size) in a transfer buffer (380 mM glycine, 50 mM Tris–HCl, 20% methanol, 1% SDS). After blocking for 1 h at room temperature with 5% non-fat dry milk in Tris-buffered saline (TTBS; 137 mM NaCl, 20 mM Tris–HCl, 0.1% Tween 20, pH 7.4), the PVDF membrane was then incubated for 180 min with polyclonal rabbit antibodies raised against MPO (1∶1000 dilution; Abcam, Cambridge, UK; catalog no. ab9535), TNF-α (1∶1000 dilution; AnaSpec, San Jose, CA, USA; catalog no. 55383), or iNOS (1∶1000 dilution; BD Pharmingen, San Diego, CA, USA; catalog no. 610332) proteins at room temperature. The MPO, TNF-α, and iNOS antibodies recognized bands at ∼84, ∼40, and ∼130 kDa, respectively. Immunoreactive bands of protein were visualized by enhanced chemiluminescence (ECL kit; Millipore), and images were acquired using the UVP BioChemi imaging system (UVP LLC, Upland, CA, USA). The relative densitometric quantification of the immunoreactive bands of MPO, TNF-α, and iNOS proteins was performed using LabWorks 4.0 software (UVP LLC, Upland, CA, USA). At the end, the PVDF membranes were reprobed with an anti-β-actin antibody (1∶2500 dilution; GeneTex, San Antonio, TX, USA; catalog no. GTX124500; polyclonal rabbit antibody). The relative variation between the bands in the vehicle and treatment group samples was calculated using the same image.

### 6. Data and statistical analysis

All data are represented as mean ± standard error of the mean (SEM). For statistical analysis, we calculated differences between the experimental groups using a one-way analysis of variance (ANOVA) followed by the Student-Newman-Keuls post-hoc test. We defined *P*<0.05 as the threshold for statistical significance.

## Results

### 1. The effect of an i.p. injection of carrageenan on the adult zebrafish

The common intraplantar dosage of carrageenan in the rat model ranges between 1% and 2% in 100 µL [6], [7], [28], [38]–[41]. In the present study using the zebrafish model, we selected a dosage range from 0.75–3% carrageenan (in a volume of 20 µL) that was based on our previous practical experience (1.5% carrageenan) in the rat model [6], [7]. We divided the adult zebrafish into 4 groups based on the concentration of carrageenan injected; i) i.p. injection with vehicle (i.e., PBS; control group); ii) i.p. injection with 0.75% carrageenan; iii) i.p. injection with 1.5% carrageenan; and iv) i.p. injection with 3% carrageenan. When we measured whole-body volume, we found that i.p. injection with vehicle did not cause significant changes in volume at any of the time points examined (up to 8 h after injection) (Fig. 1A). To simplify data analysis for statistical purposes, we calculated the area under the curve (AUC) of the whole-body-edematous effect-time curve (Fig. 1B) by the trapezoidal method [36]. AUC analysis showed that i.p. injection of 0.75–3% carrageenan induced a significant increase in the whole-body volume in adult zebrafish (Fig. 1B).

As a methodological improvement for measuring edema in carrageenan-injected adult zebrafish, we next used photographic image analysis (instead of the rat paw volume meter method) so that we were able to focus on changes in abdominal size, in order to minimize the stress within the measuring process. The photographic image analysis method was then used to determine the time course of increase in the lateral area of the abdomen induced by i.p. injection of 1.5% carrageenan. In this experiment, we divided adult zebrafish into the following 6 groups: i) i.p. injection of vehicle (PBS) at 8 h; ii) i.p. injection of vehicle at 16 h; iii) i.p. injection of PBS at 24 h; iv) i.p. injection of 1.5% carrageenan at 8 h, v) i.p. injection of 1.5% carrageenan at 16 h; and vi) i.p. injection of 1.5% carrageenan at 24 h. Among these groups, the first 3 were used as control groups and the other groups as the treatment groups. We found that there were no significant differences in the lateral area of abdomen induction after vehicle injection at 8 h, 16 h, and 24 h (Fig. 2). Conversely, the lateral area of the abdomen increased progressively and significantly in the carrageenan-treated groups after 24 h compared with the vehicle-treated groups (Fig. 2). At 24 h, i.p. carrageenan (Fig. 2K) markedly increased leukocyte infiltration in the abdominal tissues of zebrafish compared with the vehicle-treated group (Fig. 2J). Based on these results, we chose 24 h after i.p. injection as the time point for further experiments to investigate the anti-edematous effect of MP, which served as the positive control to control inflammation in the carrageenan-injected adult zebrafish.

### 2. The effect of an i.p. injection of MP on carrageenan-induced inflammation in adult zebrafish

**Figure 3:**
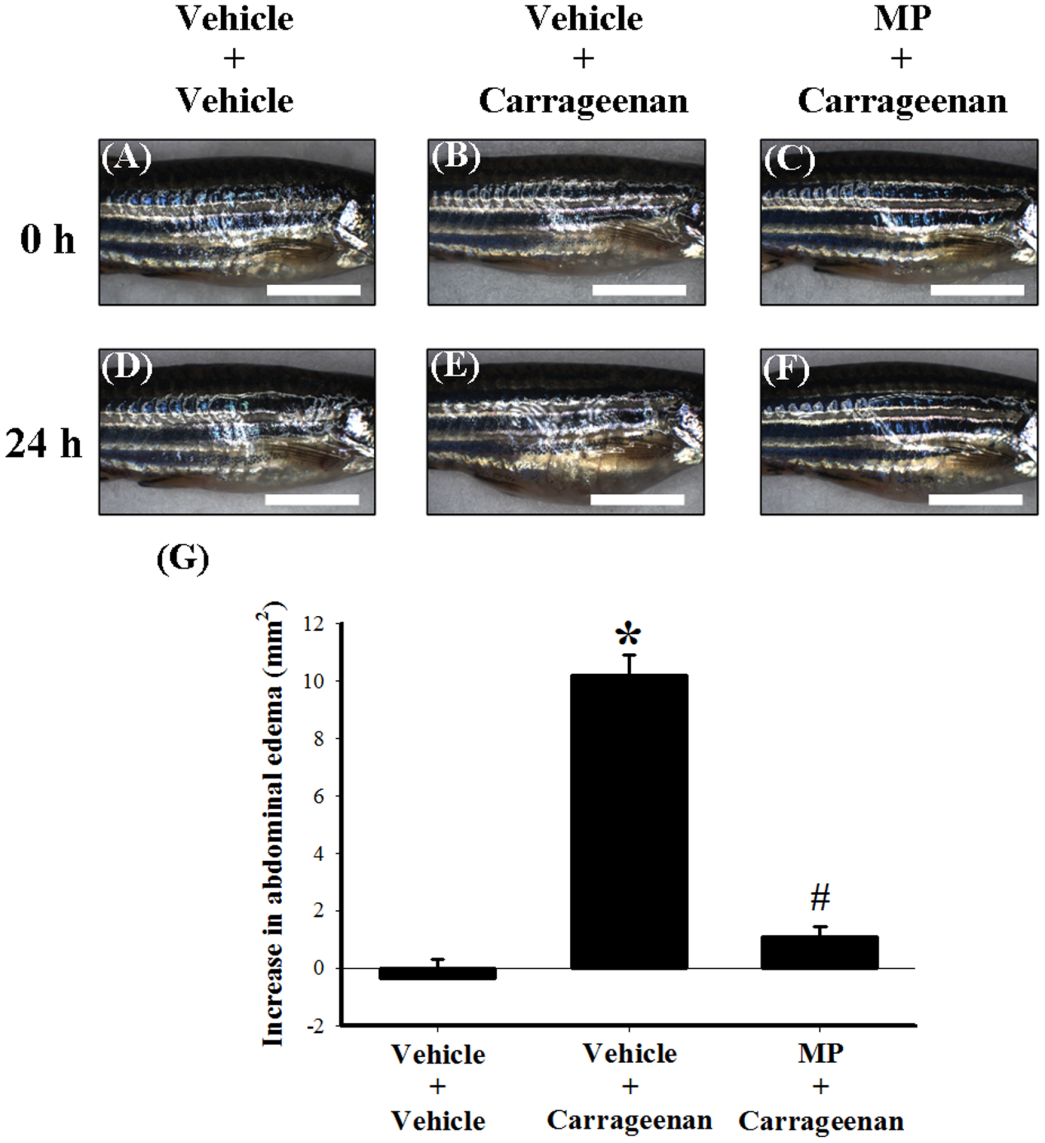
The anti-edematous effects of i.p. MP in carrageenan-injected adult zebrafish. The photographic images show the gross pathology of the abdomen in the lateral view from the i.p. injection vehicle (PBS) plus i.p. injection vehicle group (A and D), the i.p. injection vehicle plus i.p. injection 1.5% carrageenan group (B and E), and the i.p. injection MP plus i.p. 1.5% carrageenan group (C and F). Images A–C were taken at 0 h after the second injection (as well as 1 h after the first injection); images D–F were taken at 24 h after the second injection (as well as 25 h after the first vehicle or MP injection). Scale bars: 5 mm (for all images). Quantification of the lateral area of abdomen induced by i.p. injection of vehicle or carrageenan in adult zebrafish using a photographic image analysis system (G). We used 1 µg MP as a positive control. MP administered by i.p. injection 1 h before carrageenan injection significantly inhibited carrageenan-induced abdominal edema. Each bar in Figure G represents the mean ± SEM of 9 adult zebrafish per group. MP: methylprednisolone. * *P*<0.05 compared with the i.p. vehicle plus i.p. vehicle group; #*P*<0.05 compared with the i.p. vehicle plus i.p. 1.5% carrageenan group.

**Figure 4:**
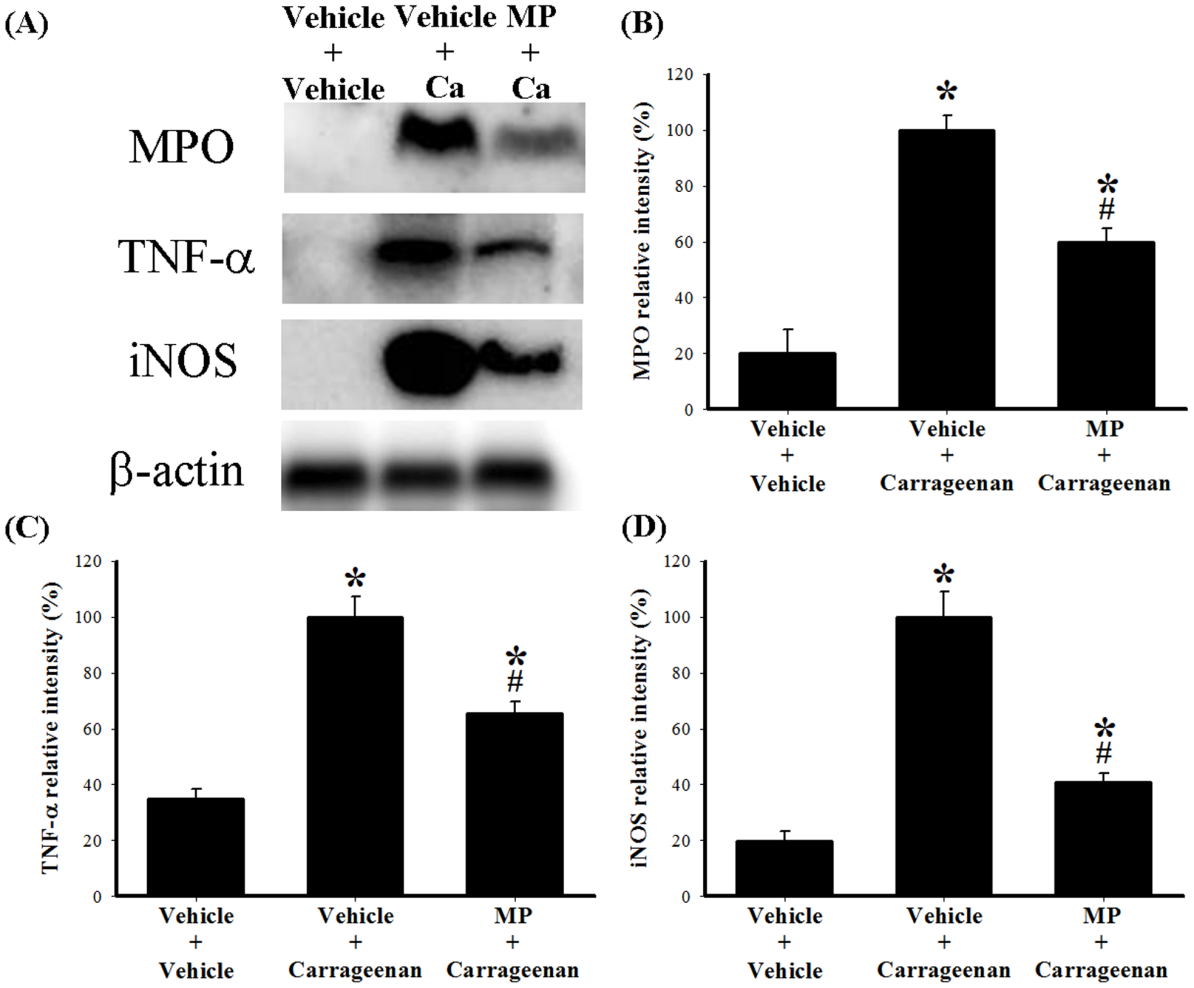
The effect of MP on protein expression of MPO, TNF-α, and iNOS in carrageenan-injected zebrafish. (A) Western blots for MPO, TNF-α, iNOS, and β-actin proteins from adult zebrafish; (B) relative density of the MPO immunoblot; (C) relative density of the TNF-α immunoblot; (D) relative density of the iNOS immunoblot. There are 3 groups: vehicle+vehicle: i.p. injection first vehicle (20 µL PBS) 1 h before i.p. second vehicle (20 µL PBS); vehicle+carra: i.p. vehicle (20 µL PBS) 1 h before i.p. 1.5% carrageenan (20 µL); MP+carra: i.p. 1 µg MP (20 µL) 1 h before i.p. 1.5% carrageenan (20 µL). The abdominal samples from adult zebrafish were collected at 24 h after the second injection (as well as 25 h after the first injection). Relative intensity of the i.p. vehicle plus i.p. 1.5% carrageenan group was taken to be 100%. Band intensities of MPO, TNF-α, and iNOS were quantified by densitometry and are indicated as a percentage change relative to that of the vehicle plus carrageenan group. Western blotting of the abdominal tissue homogenate revealed that injection of carrageenan evoked significant upregulation of MPO, TNF-α, and iNOS proteins at 24 h compared with that of the vehicle plus vehicle group. MP administered at a dose of 1 µg (i.p.) 1 h prior to i.p. 1.5% carrageenan significantly reduced carrageenan-induced upregulation of MPO, TNF-α, and iNOS. Western blotting of β-actin was performed to verify that equivalent amounts of protein were loaded in each lane. These experiments were repeated 3 times. Ca: carrageenan; MP: methylprednisolone; MPO: myeloperoxidase, TNF-α: tumor necrosis factor-α; iNOS: inducible nitric oxide synthase. * *P*<0.05 compared with the i.p. vehicle plus i.p. vehicle group; #*P*<0.05 compared with the i.p. vehicle plus i.p. 1.5% carrageenan group.

To evaluate the anti-edematous effects of an i.p. injection of MP on carrageenan-injected adult zebrafish, we used the following 3 groups: i) i.p. injection with vehicle (PBS) plus a second i.p. injection with vehicle (PBS; control group); ii) i.p. injection with vehicle plus i.p. injection with 1.5% carrageenan; and iii) i.p. injection with MP (1 µg) plus i.p. injection of 1.5% carrageenan. The volume of both injections was 20 µL, and the first injection of vehicle or MP was given 1 h before the second injection of vehicle or carrageenan. As expected, injection with vehicle followed by carrageenan (Fig. 3B and 3E) produced significant abdominal edema at 24 h compared to injection with vehicle followed by a second injection with vehicle (Fig. 3A and 3D). Compared to injection with vehicle plus injection with carrageenan (Fig. 3B and 3E), injection of MP before the carrageenan injection significantly inhibited carrageenan-induced abdominal edema (Fig. 3C and 3F). Measurements of the lateral area of the abdomen also indicated that MP exerted anti-edematous effects in the carrageenan-injected adult zebrafish (Fig. 3G). We then investigated whether carrageenan and MP affect levels of the leukocyte marker MPO and the proinflammatory proteins TNF-α and iNOS in a manner that would be consistent with the observed edematous effects of carrageenan and the anti-edematous effects of MP. Western blots were performed with the abdominal tissue homogenate; the results showed that injection with carrageenan significantly upregulated the expression of MPO, TNF-α, and iNOS proteins at 24 h compared with that of the control group (injection with vehicle followed by a second injection with vehicle) (Fig. 4A). Moreover, MP administration prior to carrageenan injection significantly reduced the levels of MPO, TNF-α, and iNOS to 59.69±5.09% (Fig. 4B), 65.29±4.54% (Fig. 4C), and 40.95±3.16% (Fig. 4D), respectively, of the levels seen in the vehicle plus carrageenan group. In addition, we confirmed the molecular specificity of the Abcam MPO antibody # ab9535 for western blot analysis (Fig. S1).

### 3. The effect of i.p. administration of an anti-TNF-α antibody and AG on carrageenan-injected adult zebrafish

**Figure 5:**
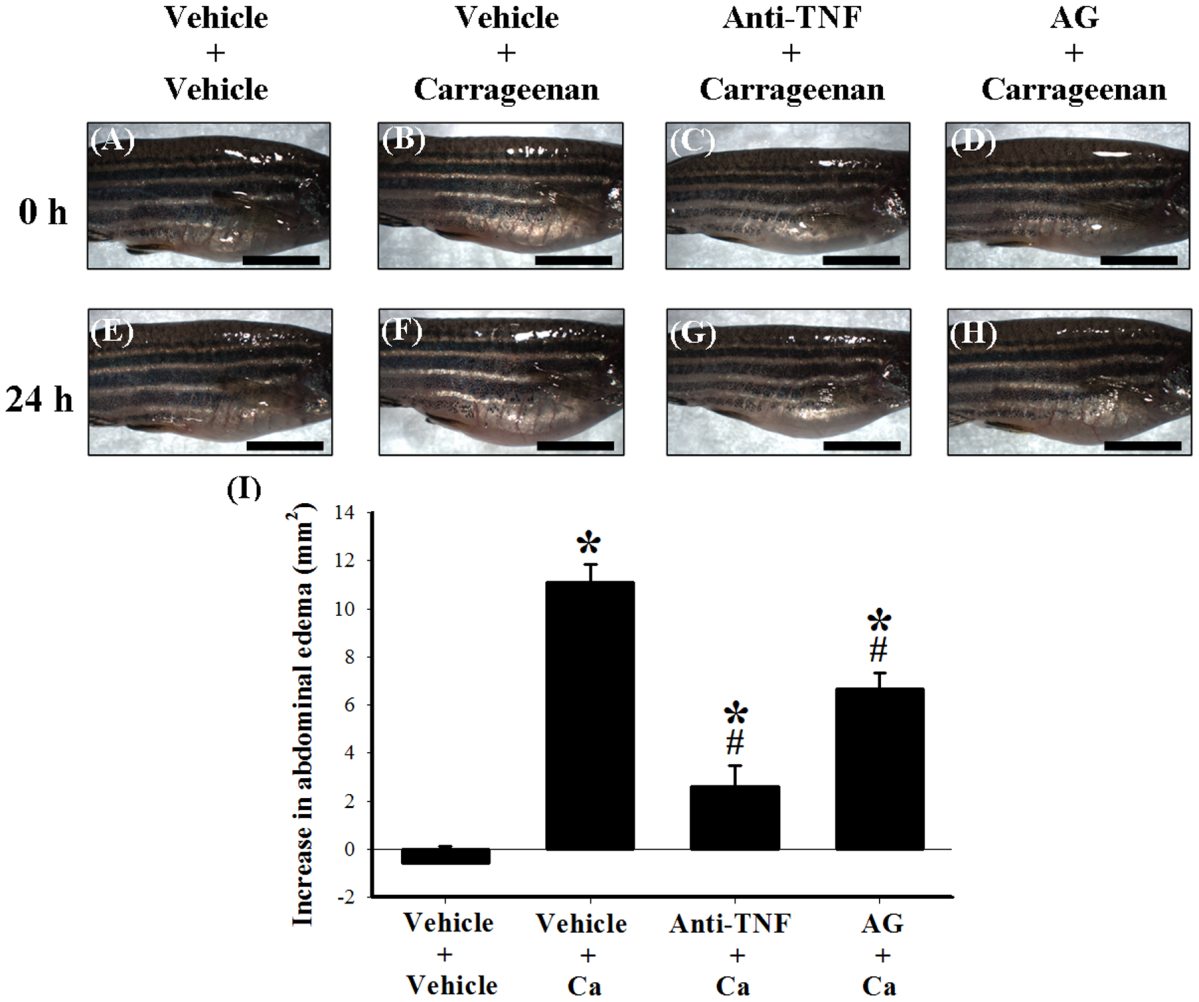
The anti-edematous effects of i.p. anti-TNF-α antibody and AG in carrageenan-injected adult zebrafish. The photographic images show the gross pathology of the abdomen in the lateral view in the i.p. vehicle (PBS) plus i.p. vehicle group (A and E), the i.p. vehicle plus i.p. 1.5% carrageenan group (B and F), the i.p. anti-TNF-α antibody (1∶5 dilution in a volume of 20 µL) plus i.p. 1.5% carrageenan group (C and G), and the i.p. AG (1 µg) plus i.p. 1.5% carrageenan group (D and H). Images A–D were taken at 0 h after the second injection of vehicle or carrageenan (as well as 1 h after the first injection of vehicle, anti-TNF-α antibody, or AG); images E–H were taken at 24 h after the second injection of vehicle or carrageenan (as well as 25 h after the first injection of vehicle, anti-TNF-α antibody, or AG injection). Scale bars: 5 mm for all images. Quantification of the lateral area of the abdomen induced by the second injection of vehicle or carrageenan in adult zebrafish using a photographic image analysis system (I). Anti-TNF-α antibody or AG administered 1 h before carrageenan injection markedly reduced carrageenan-induced abdominal edema. Each bar in Figure (I) represents the mean ± SEM of 9 adult zebrafish per group. anti-TNF: anti-TNF-α antibody; AG: aminoguanidine; Ca: carrageenan; * *P*<0.05 compared with the i.p. injection of vehicle plus i.p. vehicle group; #*P*<0.05 compared with the i.p. vehicle plus i.p. 1.5% carrageenan group.

The western blot results for TNF-α and iNOS from carrageenan-injected adult zebrafish were consistent with previous reports on carrageenan-induced inflamed paws in rodents [7], [28]–[30]; and it has been reported that systemic injection of anti-TNF-α antibody and the iNOS-selective inhibitor AG inhibited carrageenan-induced paw edema in rodents [28]–[31]. Therefore, we predicted that both the anti-TNF-α antibody and AG would produce similar anti-edematous effects in carrageenan-injected adult zebrafish. To test our hypothesis, we used the following 4 groups: i) i.p. injection with vehicle (PBS) followed by a second i.p. injection with vehicle (control group); ii) i.p. injection with vehicle followed by i.p. injection with 1.5% carrageenan; iii) i.p. injection with the anti-TNF-α antibody (1∶5 dilution in a volume of 20 µl) followed by i.p. injection with 1.5% carrageenan; and iv) i.p. injection with AG (1 µg) followed by i.p. injection with 1.5% carrageenan. Compared to injection with vehicle followed by injection with carrageenan (Fig. 5B and 5F), anti-TNF-α antibody (Fig. 5C and 5G) or AG (Fig. 5D and 5H) administered 1 h before carrageenan injection markedly reduced the carrageenan-induced abdominal edema. Measurements of the lateral abdominal area also showed the anti-edematous effects of the anti-TNF-α antibody and AG in carrageenan-injected adult zebrafish (Fig. 5I).

## Discussion

### 1. Summary of our findings

The optimization of the concentration of an induction compound is very important for the development of a successful animal model [42], [43]. In this present study, we tested different concentrations of carrageenan (0.75%, 1.5%, and 3.0%) to determine the optimum dosage. In these experiments, we used the paw volume meter to simply the measurement of zebrafish body volume. Based on these preliminary experiments, we decided to use 1.5% carrageenan to elicit edematous responses in subsequent experiments, in which we measured body edema using an image analysis method instead of the paw volume meter. In this study, we observed that i.p. injection of carrageenan produced typical symptoms of inflammation in zebrafish, such as swelling, and upregulated MPO, a leukocyte marker, as well as the proinflammatory proteins TNF-α and iNOS. Furthermore, we also demonstrated that during carrageenan-evoked edema, MPO, TNF-α, and iNOS were significantly inhibited by i.p. pretreatment with MP. Moreover, i.p. injection pretreatment with anti-TNF-α antibody or AG, an iNOS-selective inhibitor, also inhibited carrageenan-induced edema. Therefore, we conclude that the inflammatory responses of carrageenan-injected adult zebrafish can be modulated by known compounds with anti-inflammatory properties.

### 2. The proinflammatory properties of carrageenan in fish

To develop this novel inflammatory edema model using zebrafish, the gastrointestinal inflammatory compound carrageenan was used [44]. This compound was first obtained from the red alga *Chondrus crispus* in 1862 and is a high-molecular-weight sulfated polysaccharide with 3 main types of structures: ι, κ, and λ [5]. To date, very little information are available about the proinflammatory properties of carrageenan in fish compared with rodents. Much of the information on this topic concerns the immunostimulant properties of ι-carrageenan or κ-carrageenan in teleost fish against bacterial infections [45]–[47]. In contrast, local injection of λ-carrageenan into soft tissues in rodents induces acute inflammation [48]; and our previous studies on acute inflammation in the rat [6], [7] corroborate our present findings in zebrafish (Figs. 1, 2, and 4). Surprisingly, in an earlier study to understand macrophage variation in *Streptococcus pyogenes* virulence, in an attempt to establish zebrafish as an infectious disease model, when carrageenan was i.p. injected into adult zebrafish, a reduction in macrophage count was found [49]. In addition, in 1977, intramuscular injection of carrageenan was reported to induce only granuloma, a histopathological inflammatory response, in the teleost fish, plaice (*Pleuronectes platessa*) [50]. Nonetheless, these studies show that carrageenan injection can influence the immune system in fish [45]–[47], [49], [50]. Until today, no study has used carrageenan to investigate the inflammation using zebrafish. We are the first research group to explore the proinflammatory properties of carrageenan in zebrafish.

### 3. The characteristics of i.p. carrageenan in zebrafish

In this study, the inflammatory response is characterized by edema in the abdominal region. Edema formation is a result of the upregulation of proinflammatory proteins and the accumulation of leukocytes at the inflammatory site [5], [51]. At 24 h, histopathology also revealed that i.p. carrageenan induced leukocyte infiltration in the abdominal tissues of zebrafish (Fig. 2K). However, up until now, there was no information available about the expression of the leukocyte marker MPO [52]–[54] and the proinflammatory proteins TNF-α and iNOS [55] during inflammatory conditions in a zebrafish model. We found that abdominal edema accompanied the upregulation of MPO, TNF-α, and iNOS in adult zebrafish at 24 h after i.p. injection with 1.5% carrageenan (Fig. 4), which is consistent with above-mentioned characteristics of the inflammatory response. In addition, immunohistochemistry showed that i.p. carrageenan markedly increased iNOS immunoreactivity in the abdominal tissues of zebrafish, especially in the intestine (Fig. S2). Moreover, i.p. pretreatment with anti-TNF-α antibody or the iNOS-selective inhibitor AG inhibited carrageenan-induced abdominal edema in adult zebrafish (Fig. 5). The anti-edematous effects of anti-TNF-α antibody and AG on carrageenan-injected adult zebrafish, which are similar with their anti-edematous effects in carrageenan-induced paw edema in rodents [28]–[31], suggest that TNF-α and iNOS play key roles in the edematous process in carrageenan-injected adult zebrafish. In the current study, we used commercial antibodies for zebrafish, although antibody tools for zebrafish are still insufficient compared with the complete antibody system for rodent model [56]. Therefore, we also hope that our present study will remind scientists and commercial antibody companies to pay more attention to the development of antibody tools for zebrafish, which will inspire researchers to perform further studies in the field of immunology using this valuable model organism.

### 4. The possible molecular index for screening compounds with anti-inflammatory activity in zebrafish

Several bioactive compounds or antibodies have been examined in rodent models, but most of them have not been tested in the zebrafish model yet. Like AG and the anti-TNF-α antibody [28]–[31], systemic corticosteroids such as MP are known to be inhibitors of carrageenan-induced rodent paw edema and inflammation [57]. In addition, the anti-inflammatory effects of corticosteroid (flumethasone) have been reported previously in a zebrafish model of inflammation [22]. Our experimental results showed that MP could inhibit carrageenan-induced abdominal edema (Fig. 3) and downregulate MPO, TNF-α, and iNOS at the protein level (Fig. 4), which is similar to the effect of MP in carrageenan-induced paw edema in rodents [57]. Together, our results showing the anti-edematous effects of MP, anti-TNF-α antibody, and AG support the hypothesis that zebrafish and rodents share basic inflammatory responses, which could be modulated by bioactive compounds, and that therefore the zebrafish model is suitable for use in the investigation of biomedical issues, especially in immunology [13], [14], [17], [22], [24]. Moreover, this study also confirms the ability of this model to identify anti-inflammatory compounds, and it would further be expected that novel compounds might be identified via this zebrafish model of inflammation. In summary, the upregulation of TNF-α and iNOS after i.p. carrageenan injection appears to reflect the level of tissue inflammatory response in adult zebrafish.

### 5. Advantages and novelty of carrageenan-injected adult zebrafish as an inflammation model

Carrageenan-induced edema induced in the adult zebrafish (Fig. 1 and 2) was found to follow a time pattern similar to that seen in the rodents [4]–[12]. Our carrageenan-induced adult zebrafish abdominal edema model offers several advantages over equivalent rodent models (Table 1). With the zebrafish model, it is possible to screen anti-inflammatory compounds using lower dosages than possible with the rodent model. For example, the common systemic dosage required to obtain the anti-edematous effects of MP and AG is at the mg level for a 300 g rat [28], [57] but is only at µg levels for an adult zebrafish. Another advantage of the carrageenan-injected adult zebrafish model is that it is easier to replicate experiments and more economical to evaluate the toxic effects (swelling responses, death, or external side effects) of the compound than when using a rodent model. The zebrafish model that we have established here differs from the existing zebrafish inflammation models in 3 important aspects. First, our model, with adult zebrafish, is able to address both innate and adaptative responses, whereas most of the other studies, with larvae or embryos, deal with only one type of immune response (the innate immune response) [13]. Second, we address the phenomenon of “swelling”, a typical external symptom of inflammation, as an assessment in a zebrafish model. The methodology of the photographic image analysis method would be useful for studying the edematous effects of bioactive compounds, if any, as part of safety testing. And third, using our zebrafish model reduces the difficulty of the experimental procedure itself, which is an improvement in experimental design in general. However, certainly, the existing inflammation models using the larval or embryonic stages of zebrafish are more suitable for higher-throughput screening than our model using adult zebrafish. The present study provides a novel adult zebrafish abdominal edema model that can be used as an ideal tool to bridge the gap between the traditional *in vitro* systems or the existing *in vivo* zebrafish inflammation models (using the larval or embryonic stages) and *in vivo* rodent systems in basic research for identifying novel therapeutics for inflammation-related diseases. A more refined and precise experimental design of rodent studies can be achieved on the basis of the *in vivo* data about edema obtained from carrageenan-injected adult zebrafish.

**Table 1:** Comparison of the carrageenan-injected adult zebrafish model to the carrageenan-injected rodent model.

Item	Carrageenan-injected adult zebrafish	Carrageenan-injected rodent model	References of rodents
Edema	Yes	Yes	[4]–[12]
MPO ↑(Leukocyte marker)	Yes	Yes	[6], [7], [54]
Upregulation of TNF-α at protein level	Yes	Yes	[29], [30]
Upregulation of iNOS at protein level	Yes	Yes	[7], [28]
Edema could be modulated by bioactive compounds	Yes	Yes	[28]–[31], [57]
The dosage to test compounds	At µg level	At mg level	[28], [57]

### 6. Conclusions

Our results show that i.p. administration of carrageenan induces significant abdominal edema, which is accompanied by changes in the expression of MPO, TNF-α, and iNOS at the protein level and which was significant inhibited by i.p. administration of MP. The present study provides a novel *in vivo* zebrafish inflammatory edema model for the screening of small volumes of drugs or compounds with anti-inflammatory activity.

## Supporting Information

Figure S1 **The molecular specificity of the Abcam MPO antibody #ab9535 for western blot analysis.** We used recombinant human MPO (R&D Systems, Minneapolis, MN, USA; catalog no. 3174-MP) as the positive control. The abdominal samples from adult zebrafish were collected at 24 h after the second injection (as well as at 25 h after the first injection). There are 3 groups: vehicle+vehicle: i.p. injection first vehicle (20 µL PBS) 1 h before i.p. second vehicle (20 µL PBS); recombinant human MPO; vehicle+carra: i.p. vehicle (20 µL PBS) 1 h before i.p. 1.5% carrageenan (20 µL). Western blotting revealed that the Abcam MPO antibody #ab9535 could detect recombinant human MPO-evoked as well as carrageenan-evoked significant upregulation of MPO. These experiments were repeated 3 times. Ca: carrageenan; MPO: myeloperoxidase.
(TIF)

Figure S2 **Upregulatory effect of carrageenan on iNOS protein expression in abdominal tissues of zebrafish.** For immunohistochemistry, after deparaffinization in xylene and rehydration with a graded series of ethanol, endogenous peroxidase activity of the abdominal sections was quenched using 0.3% H_2_O_2_ for 30 min. Then, the sections were permeabilized with 0.1% Triton X-100 in PBS for 20 min. Following retrieval of the antigen with proteinase K (20 mM; Sigma) in PBS for 20 min, to decrease nonspecific adsorption we incubated the sections using 5% normal goat serum in PBS for 30 min. The sections were incubated overnight at 4°C with anti-iNOS (1∶100 dilution; BD Pharmingen, San Diego, CA, USA; catalog no. 610332) antibody. Finally, after incubation with biotin-conjugated anti-rabbit IgG (1∶200 dilution; Vector Laboratories Inc, Burlingame, CA, USA; catalog no. BA-1100) for 30 min followed by avidin-biotin-peroxidase complex for 30 min (Vectastain ABC kit; Vector Laboratories Inc, Burlingame, CA, USA; catalog no. PK-6100), the sections were incubated with 3,3′-diaminobenzidine tetrahydrochloride (DAB) (Vectastain ABC kit; Vector Laboratories Inc, Burlingame, CA, USA; catalog no. SK-4100) for 8 min. We analyzed the all stained sections using a Leica DM-6000 CS microscope (Leica Instruments Inc., Wetzlar, Germany) and a microscope digital camera system (SPOT Idea 5 MP CMOS scientific color digital camera system, Diagnostic Instruments, Inc., Sterling Heights, MI, USA). The sections (2 µm) at 24 h after an i.p. injection of vehicle (A) or carrageenan (B). I.p. carrageenan obviously increased iNOS immunoreactivity of the intestine. Scale bars: 100 µm for all images.
(TIF)

## References

[1] Ferrero-Miliani L, Nielsen OH, Andersen PS, Girardin SE (2007) Chronic inflammation: importance of NOD2 and NALP3 in interleukin-1beta generation. Clin Exp Immunol 147: 227–235. 17223962 10.1111/j.1365-2249.2006.03261.x PMC1810472

[2] Martin P, Leibovich SJ (2005) Inflammatory cells during wound repair: the good, the bad and the ugly. Trends Cell Biol 15: 599–607. 16202600 10.1016/j.tcb.2005.09.002

[3] Renkin EM (1994) Cellular aspects of transvascular exchange: a 40-year perspective. Microcirculation 1: 157–167. 8790586 10.3109/10739689409148270

[4] Winter CA, Risley EA, Nuss GW (1962) Carrageenin-induced edema in hind paw of the rat as an assay for antiiflammatory drugs. Proc Soc Exp Biol Med 111: 544–547. 14001233 10.3181/00379727-111-27849

[5] Morris CJ (2003) Carrageenan-Induced Paw Edema in the Rat and Mouse. In: Winyard PG, Willoughby DA, editors. Inflammation Protocols: Humana Press. pp. 115–121.

[6] Huang SY, Chen NF, Chen WF, Hung HC, Lee HP, et al (2012) Sinularin from indigenous soft coral attenuates nociceptive responses and spinal neuroinflammation in carrageenan-induced inflammatory rat model. Mar Drugs 10: 1899–1919. 23118711 10.3390/md10091899 PMC3475263

[7] Jean YH, Chen WF, Duh CY, Huang SY, Hsu CH, et al (2008) Inducible nitric oxide synthase and cyclooxygenase-2 participate in anti-inflammatory and analgesic effects of the natural marine compound lemnalol from Formosan soft coral *Lemnalia cervicorni* . Eur J Pharmacol 578: 323–331. 17916350 10.1016/j.ejphar.2007.08.048

[8] Levy L (1969) Carrageenan paw edema in the mouse. Life Sci 8: 601–606. 10.1016/0024-3205(69)90021-6 5799282

[9] Backhouse N, Delporte C, Givernau M, Cassels BK, Valenzuela A, et al (1994) Anti-inflammatory and antipyretic effects of boldine. Agents Actions 42: 114–117. 7879695 10.1007/BF01983475

[10] Backhouse N, Delporte C, Negrete R, Feliciano SA, Lopez-Perez JL (2002) Bioactive phenolic derivatives from *Acaena splendens* methanol extract. Phytother Res 16: 562–566. 12237815 10.1002/ptr.997

[11] Delporte C, Munoz O, Rojas J, Ferrandiz M, Paya M, et al (2002) Pharmaco-toxicological study of *Kageneckia oblonga*, Rosaceae. Z Naturforsch C 57: 100–108. 11926521 10.1515/znc-2002-1-218

[12] Backhouse N, Delporte C, Negrete R, Salinas P, Pinto A, et al (1996) Antiinflammatory and antipyretic activities of *Cuscuta chilensis*, *Cestrum parqui* and *Psoralea glandulosa* . Int J Pharmacogn 34: 53–57.

[13] Novoa B, Figueras A (2012) Zebrafish: model for the study of inflammation and the innate immune response to infectious diseases. Adv Exp Med Biol 946: 253–275. 21948373 10.1007/978-1-4614-0106-3_15

[14] d'Alencon C, Pena O, Wittmann C, Gallardo V, Jones R, et al (2010) A high-throughput chemically induced inflammation assay in zebrafish. BMC Biol 8: 151. 21176202 10.1186/1741-7007-8-151 PMC3022775

[15] Chakraborty C, Hsu CH, Wen ZH, Lin CS, Agoramoorthy G (2009) Zebrafish: a complete animal model for *in vivo* drug discovery and development. Curr Drug Metab 10: 116–124. 19275547 10.2174/138920009787522197

[16] Hsu CH, Wen ZH, Lin CS, Chakraborty C (2007) The zebrafish model: use in studying cellular mechanisms for a spectrum of clinical disease entities. Curr Neurovasc Res 4: 111–120. 17504209 10.2174/156720207780637234

[17] Renshaw S, Ingham P (2010) Zebrafish models of the immune response: taking it on the ChIn. BMC Biol 8: 148. 21176245 10.1186/1741-7007-8-148 PMC3008691

[18] Brown SB, Tucker CS, Ford C, Lee Y, Dunbar DR, et al (2007) Class III antiarrhythmic methanesulfonanilides inhibit leukocyte recruitment in zebrafish. J Leukoc Biol 82: 79–84. 17431092 10.1189/jlb.0107030

[19] Pase L, Nowell CJ, Lieschke GJ (2012) *In vivo* real-time visualization of leukocytes and intracellular hydrogen peroxide levels during a zebrafish acute inflammation assay. Methods Enzymol 506: 135–156. 22341223 10.1016/B978-0-12-391856-7.00032-9

[20] Park KH, Cho KH (2011) A zebrafish model for the rapid evaluation of pro-oxidative and inflammatory death by lipopolysaccharide, oxidized low-density lipoproteins, and glycated high-density lipoproteins. Fish Shellfish Immunol 31: 904–910. 21906681 10.1016/j.fsi.2011.08.006

[21] Fleming A, Jankowski J, Goldsmith P (2010) *In vivo* analysis of gut function and disease changes in a zebrafish larvae model of inflammatory bowel disease: a feasibility study. Inflamm Bowel Dis 16: 1162–1172. 20128011 10.1002/ibd.21200

[22] Loynes CA, Martin JS, Robertson A, Trushell DMI, Ingham PW, et al (2010) Pivotal Advance: Pharmacological manipulation of inflammation resolution during spontaneously resolving tissue neutrophilia in the zebrafish. J Leukoc Biol 87: 203–212. 19850882 10.1189/jlb.0409255 PMC2812557

[23] Mathew LK, Sengupta S, Kawakami A, Andreasen EA, Lohr CV, et al (2007) Unraveling tissue regeneration pathways using chemical genetics. J Biol Chem 282: 35202–35210. 17848559 10.1074/jbc.M706640200

[24] Renshaw SA, Loynes CA, Trushell DM, Ingham PW, Whyte MB (2006) The molecular controls of resolution of inflammation: what can we learn from zebrafish? Eur Respir Rev 15: 168–169.

[25] Oehlers SH, Flores MV, Hall CJ, Okuda KS, Sison JO, et al (2013) Chemically induced intestinal damage models in zebrafish larvae. Zebrafish 10: 184–193. 23448252 10.1089/zeb.2012.0824

[26] Tobin DM, Roca FJ, Oh SF, McFarland R, Vickery TW, et al (2012) Host genotype-specific therapies can optimize the inflammatory response to mycobacterial infections. Cell 148: 434–446. 22304914 10.1016/j.cell.2011.12.023 PMC3433720

[27] Oehlers SH, Flores MV, Okuda KS, Hall CJ, Crosier KE, et al (2011) A chemical enterocolitis model in zebrafish larvae that is dependent on microbiota and responsive to pharmacological agents. Dev Dyn 240: 288–298. 21181946 10.1002/dvdy.22519

[28] Salvemini D, Wang ZQ, Wyatt PS, Bourdon DM, Marino MH, et al (1996) Nitric oxide: a key mediator in the early and late phase of carrageenan-induced rat paw inflammation. Br J Pharmacol 118: 829–838. 8799551 10.1111/j.1476-5381.1996.tb15475.x PMC1909531

[29] Nishikori T, Irie K, Suganuma T, Ozaki M, Yoshioka T (2002) Anti-inflammatory potency of FR167653, a p38 mitogen-activated protein kinase inhibitor, in mouse models of acute inflammation. Eur J Pharmacol 451: 327–333. 12242095 10.1016/s0014-2999(02)02238-0

[30] Chen YL, Le Vraux V, Giroud JP, Chauvelot-Moachon L (1994) Anti-tumor necrosis factor properties of non-peptide drugs in acute-phase responses. Eur J Pharmacol 271: 319–327. 7705432 10.1016/0014-2999(94)90789-7

[31] Rocha AC, Fernandes ES, Quintao NL, Campos MM, Calixto JB (2006) Relevance of tumour necrosis factor-alpha for the inflammatory and nociceptive responses evoked by carrageenan in the mouse paw. Br J Pharmacol 148: 688–695. 16702985 10.1038/sj.bjp.0706775 PMC1751874

[32] McDaniel JC, Massey K, Nicolaou A (2011) Fish oil supplementation alters levels of lipid mediators of inflammation in microenvironment of acute human wounds. Wound Repair Regen 19: 189–200. 21362086 10.1111/j.1524-475X.2010.00659.x PMC3686090

[33] Moor AN, Vachon DJ, Gould LJ (2009) Proteolytic activity in wound fluids and tissues derived from chronic venous leg ulcers. Wound Repair Regen 17: 832–839. 19903304 10.1111/j.1524-475X.2009.00547.x

[34] Kasuga K, Yang R, Porter TF, Agrawal N, Petasis NA, et al (2008) Rapid appearance of resolvin precursors in inflammatory exudates: novel mechanisms in resolution. J Immunol 181: 8677–8687. 19050288 10.4049/jimmunol.181.12.8677 PMC2664686

[35] Westerfield M (2000) The zebrafish book. A guide for the laboratory use of zebrafish (*Danio rerio*). Eugene, OR, USA: University of Oregon.

[36] Rowland M, Tozer TN (1995) Assessment of AUC. In: Balado D, editor. Clinical Pharmacokinetics: Concepts and Applications. Third ed. Philadelphia: Lippincott Williams and Wilkins. pp. 469–470.

[37] Lowry OH, Rosebrough NJ, Farr AL, Randall RJ (1951) Protein measurement with the Folin phenol reagent. J Biol Chem 193: 265–275. 14907713

[38] Xu Q, Fitzsimmons B, Steinauer J, O'Neill A, Newton AC, et al (2011) Spinal phosphinositide 3-kinase-Akt-mammalian target of rapamycin signaling cascades in inflammation-induced hyperalgesia. J Neurosci 31: 2113–2124. 21307248 10.1523/JNEUROSCI.2139-10.2011 PMC3097059

[39] Seibert K, Zhang Y, Leahy K, Hauser S, Masferrer J, et al (1994) Pharmacological and biochemical demonstration of the role of cyclooxygenase 2 in inflammation and pain. Proc Natl Acad Sci U S A 91: 12013–12017. 7991575 10.1073/pnas.91.25.12013 PMC45366

[40] Sekiguchi F, Mita Y, Kamanaka Y, Kawao N, Matsuya H, et al (2004) The potent inducible nitric oxide synthase inhibitor ONO-1714 inhibits neuronal NOS and exerts antinociception in rats. Neurosci Lett 365: 111–115. 15245789 10.1016/j.neulet.2004.04.069

[41] Coruzzi G, Adami M, Guaita E, de Esch IJ, Leurs R (2007) Antiinflammatory and antinociceptive effects of the selective histamine H4-receptor antagonists JNJ7777120 and VUF6002 in a rat model of carrageenan-induced acute inflammation. Eur J Pharmacol 563: 240–244. 17382315 10.1016/j.ejphar.2007.02.026

[42] Small DL, Buchan AM (2000) Animal models. Br Med Bull 56: 307–317. 11092082 10.1258/0007142001903238

[43] Tomassoni D, Amenta F, Amantini C, Farfariello V, Di Cesare Mannelli L, et al (2013) Brain activity of thioctic acid enantiomers: *in vitro* and *in vivo* studies in an animal model of cerebrovascular injury. Int J Mol Sci 14: 4580–4595. 23443159 10.3390/ijms14034580 PMC3634420

[44] Tobacman JK (2001) Review of harmful gastrointestinal effects of carrageenan in animal experiments. Environ Health Perspect 109: 983–994. 11675262 10.1289/ehp.01109983 PMC1242073

[45] Cheng AC, Tu CW, Chen YY, Nan FH, Chen JC (2007) The immunostimulatory effects of sodium alginate and iota-carrageenan on orange-spotted grouper *Epinephelus coicoides* and its resistance against *Vibrio alginolyticus* . Fish Shellfish Immunol 22: 197–205. 16784873 10.1016/j.fsi.2006.04.009

[46] Fujiki K, Shin DH, Nakao M, Yano T (1997) Protective effect of kappa-carrageenan against bacterial infections in carp *Cyprinus carpio* . J Fac Agr Kyushu U 42: 113–119.

[47] Fujiki K, Shin DH, Nakao M, Yano T (1997) Effects of kappa-carrageenan on the non-specific defense system of carp *Cyprinus carpio* . Fisheries Sci 63: 934–938.

[48] Chan CC (2003) *In Vivo* Assays for COX-2. In: Winyard PG, Willoughby DA, editors. Inflammation Protocols: Humana Press. pp. 321–328.

[49] Phelps HA, Neely MN (2007) SalY of the *Streptococcus pyogenes* lantibiotic locus is required for full virulence and intracellular survival in macrophages. Infect Immun 75: 4541–4551. 17576754 10.1128/IAI.00518-07 PMC1951192

[50] Timur M, Roberts RJ (1977) Carrageenin granuloma in the plaice (*Pleuronectes platessa*); a histopathological study of chronic inflammation in a teleost fish. J Comp Pathol 87: 89–96. 838907 10.1016/0021-9975(77)90083-4

[51] Posadas I, Bucci M, Roviezzo F, Rossi A, Parente L, et al (2004) Carrageenan-induced mouse paw oedema is biphasic, age-weight dependent and displays differential nitric oxide cyclooxygenase-2 expression. Br J Pharmacol 142: 331–338. 15155540 10.1038/sj.bjp.0705650 PMC1574940

[52] Palic D, Andreasen CB, Ostojic J, Tell RM, Roth JA (2007) Zebrafish (*Danio rerio*) whole kidney assays to measure neutrophil extracellular trap release and degranulation of primary granules. J Immunol Methods 319: 87–97. 17208252 10.1016/j.jim.2006.11.003

[53] Clay H, Davis JM, Beery D, Huttenlocher A, Lyons SE, et al (2007) Dichotomous role of the macrophage in early *Mycobacterium marinum* infection of the zebrafish. Cell Host Microbe 2: 29–39. 18005715 10.1016/j.chom.2007.06.004 PMC3115716

[54] Gill N, Bijjem KR, Sharma PL (2013) Anti-inflammatory and anti-hyperalgesic effect of all-trans retinoic acid in carrageenan-induced paw edema in Wistar rats: involvement of peroxisome proliferator-activated receptor-beta/delta receptors. Indian J Pharmacol 45: 278–282. 23833373 10.4103/0253-7613.111944 PMC3696301

[55] Shin DH, Lim HS, Cho SK, Lee HY, Lee HW, et al (2000) Immunocytochemical localization of neuronal and inducible nitric oxide synthase in the retina of zebrafish, *Brachydanio rerio* . Neurosci Lett 292: 220–222. 11018316 10.1016/s0304-3940(00)01407-5

[56] Lieschke GJ, Currie PD (2007) Animal models of human disease: zebrafish swim into view. Nat Rev Genet 8: 353–367. 17440532 10.1038/nrg2091

[57] Panusa A, Selmin F, Rossoni G, Carini M, Cilurzo F, et al (2011) Methylprednisolone-loaded PLGA microspheres: a new formulation for sustained release via intra-articular administration. A comparison study with methylprednisolone acetate in rats. J Pharm Sci 100: 4580–4586. 21850665 10.1002/jps.22722

