# Supplementary figures and images for: A Novel Zebrafish Model to Provide Mechanistic Insights into the Inflammatory Events in Carrageenan-Induced Abdominal Edema
Source: PLoS One. 2014 Aug 20;9(8):e104414. doi: 10.1371/journal.pone.0104414 (PMC4139260; doi:10.1371/journal.pone.0104414)

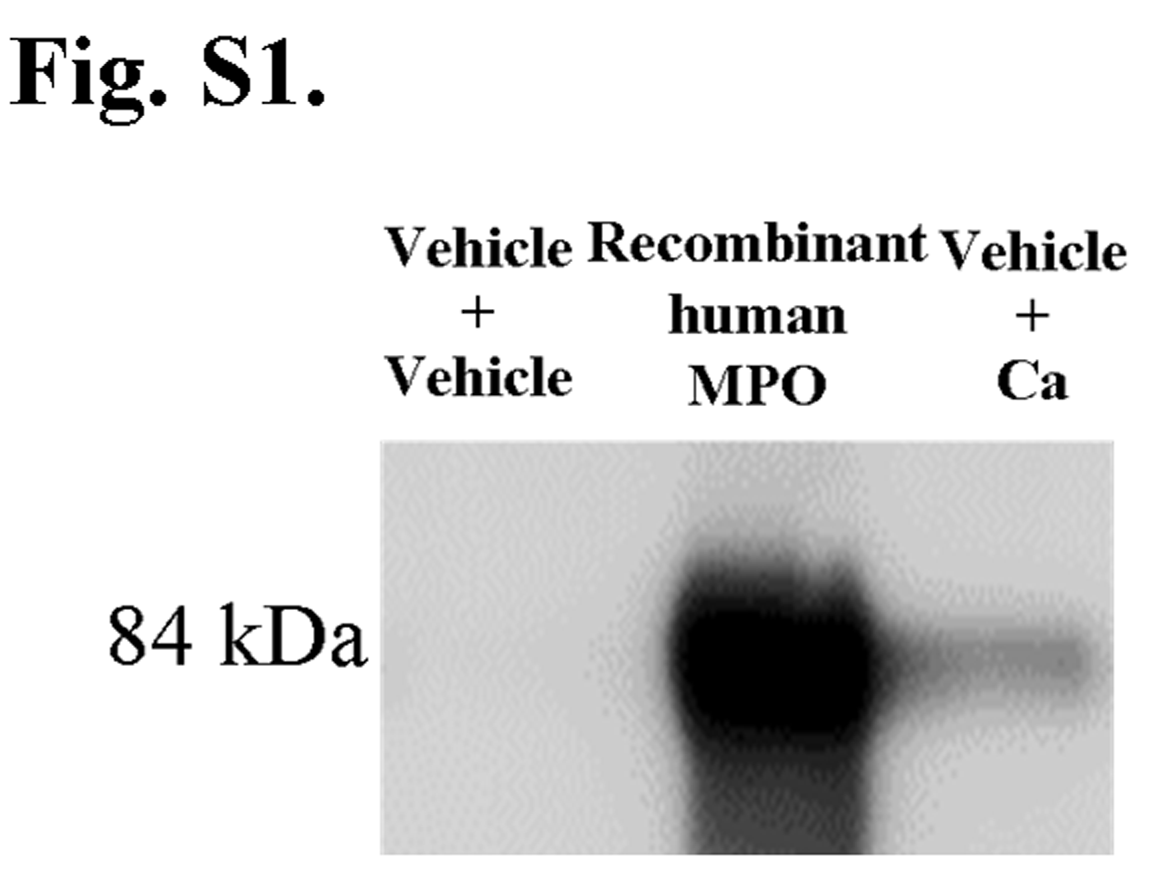

Supplement: Figure S1 — The molecular specificity of the Abcam MPO antibody #ab9535 for western blot analysis. We used recombinant human MPO (R&D Systems, Minneapolis, MN, USA; catalog no. 3174-MP) as the positive control. The abdominal samples from adult zebrafish were collected at 24 h after the second injection (as well as at 25 h after the first injection). There are 3 groups: vehicle+vehicle: i.p. injection first vehicle (20 µL PBS) 1 h before i.p. second vehicle (20 µL PBS); recombinant human MPO; vehicle+carra: i.p. vehicle (20 µL PBS) 1 h before i.p. 1.5% carrageenan (20 µL). Western blotting revealed that the Abcam MPO antibody #ab9535 could detect recombinant human MPO-evoked as well as carrageenan-evoked significant upregulation of MPO. These experiments were repeated 3 times. Ca: carrageenan; MPO: myeloperoxidase. (TIF) [file pone.0104414.s001.tif]

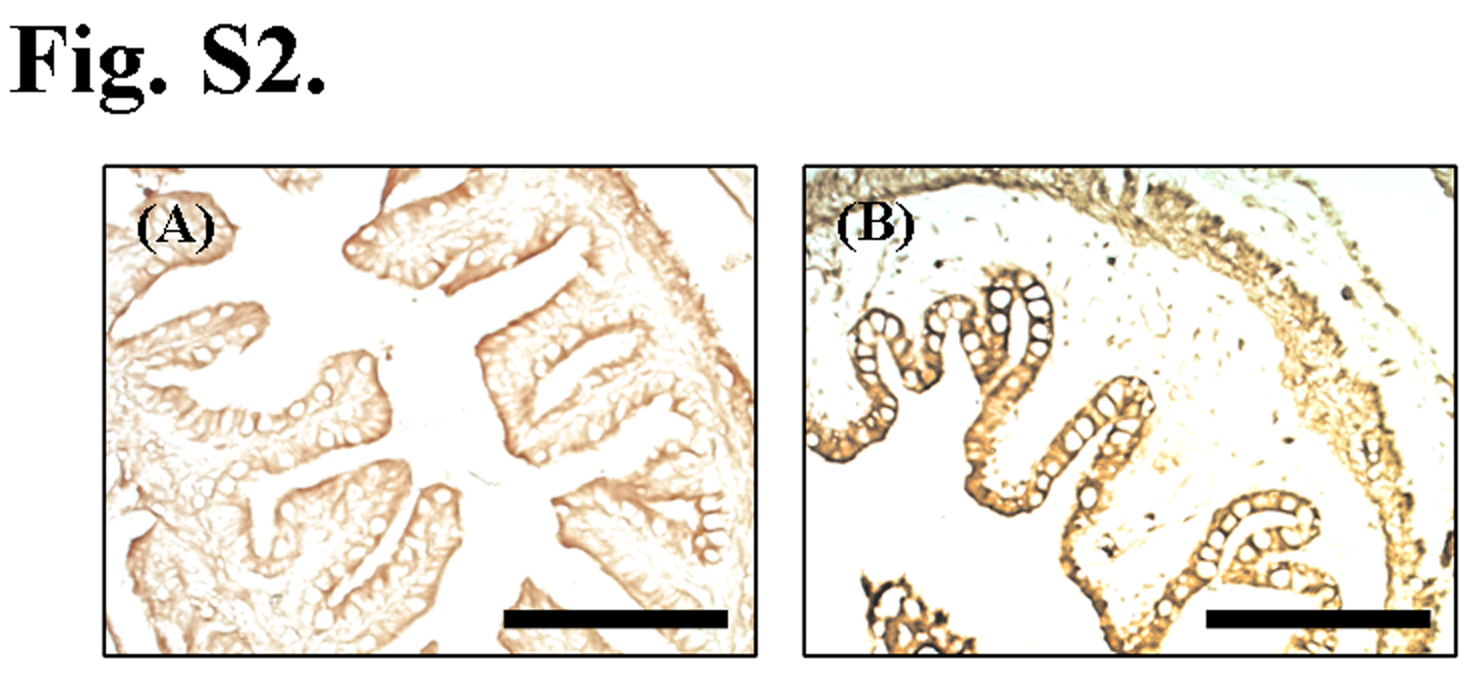

Supplement: Figure S2 — Upregulatory effect of carrageenan on iNOS protein expression in abdominal tissues of zebrafish. For immunohistochemistry, after deparaffinization in xylene and rehydration with a graded series of ethanol, endogenous peroxidase activity of the abdominal sections was quenched using 0.3% H2O2 for 30 min. Then, the sections were permeabilized with 0.1% Triton X-100 in PBS for 20 min. Following retrieval of the antigen with proteinase K (20 mM; Sigma) in PBS for 20 min, to decrease nonspecific adsorption we incubated the sections using 5% normal goat serum in PBS for 30 min. The sections were incubated overnight at 4°C with anti-iNOS (1∶100 dilution; BD Pharmingen, San Diego, CA, USA; catalog no. 610332) antibody. Finally, after incubation with biotin-conjugated anti-rabbit IgG (1∶200 dilution; Vector Laboratories Inc, Burlingame, CA, USA; catalog no. BA-1100) for 30 min followed by avidin-biotin-peroxidase complex for 30 min (Vectastain ABC kit; Vector Laboratories Inc, Burlingame, CA, USA; catalog no. PK-6100), the sections were incubated with 3,3′-diaminobenzidine tetrahydrochloride (DAB) (Vectastain ABC kit; Vector Laboratories Inc, Burlingame, CA, USA; catalog no. SK-4100) for 8 min. We analyzed the all stained sections using a Leica DM-6000 CS microscope (Leica Instruments Inc., Wetzlar, Germany) and a microscope digital camera system (SPOT Idea 5 MP CMOS scientific color digital camera system, Diagnostic Instruments, Inc., Sterling Heights, MI, USA). The sections (2 µm) at 24 h after an i.p. injection of vehicle (A) or carrageenan (B). I.p. carrageenan obviously increased iNOS immunoreactivity of the intestine. Scale bars: 100 µm for all images. (TIF) [file pone.0104414.s002.tif]
